# Metabolomics Reveals Antioxidant Metabolites in Colored Rice Grains

**DOI:** 10.3390/metabo14020120

**Published:** 2024-02-11

**Authors:** Jinyan Zhu, Ruizhi Wang, Yu Zhang, Yanyao Lu, Shuo Cai, Qiangqiang Xiong

**Affiliations:** 1 Jiangsu Key Laboratory of Crop Genetics and Physiology/Jiangsu Key Laboratory of Crop Cultivation and Physiology, Agricultural College of Yangzhou University, Yangzhou 225009, China; 006682@yzu.edu.cn (J.Z.);; 2 Jiangsu Co-Innovation Center for Modern Production Technology of Grain Crops, Yangzhou University, Yangzhou 225009, China; 3 Jiangxi Irrigation Experiment Central Station, Nanchang 330201, China

**Keywords:** colored rice, metabolomics, flavonoids, antioxidant activity

## Abstract

Colored rice is richer in nutrients and contains more nutrients and bioactive substances than ordinary white rice. Moderate consumption of black (purple) rice has a variety of physiological effects, such as antioxidant effects, blood lipid regulation, and blood sugar control. Therefore, we utilized nontargeted metabolomics, quantitative assays for flavonoid and phenolic compounds, and physiological and biochemical data to explore the correlations between metabolites and the development of antioxidant characteristics in pigmented rice seeds. The findings indicated that, among Yangjinnuo 818 (YJN818), Hongnuo (HN), Yangchannuo 1 hao (YCN1H), and Yangzi 6 hao (YZ6H), YZ6H exhibited the highest PAL activity, which was 2.13, 3.08, and 3.25 times greater than those of YJN818, HN, and YCN1H, respectively. YZ6H likewise exhibited the highest flavonoid content, which was 3.8, 7.06, and 35.54 times greater than those of YJN818, HN, and YCN1H, respectively. YZ6H also had the highest total antioxidant capacity, which was 2.42, 3.76, and 3.77 times greater than those of YJN818, HN, and YCN1H, respectively. Thus, purple rice grains have stronger antioxidant properties than other colored rice grains. Receiver operating characteristic (ROC) curve analysis revealed that trans-3,3′,4′,5,5′,7-hexahydroxyflavanone, phorizin, and trilobatin in the YZ6H, HN, and YCN1H comparison groups all had area under the curve (AUC) values of 1. Phlorizin, trans-3,3′,4′,5,5′,7-hexahydroxyflavanone, and trilobatin were recognized as indices of antioxidant capability in colored rice in this research. This research adds to the understanding of antioxidant compounds in pigmented rice, which can increase the nutritional value of rice and promote the overall well-being of individuals. This type of information is of immense importance in maintaining a balanced and healthy diet.

## 1. Introduction

Colored rice is rich in various nutrients and has various health benefits; thus, it has gradually gained widespread attention from researchers in recent years. Due to the different types and contents of anthocyanins in rice grains, brown rice can have different colors. According to the color of the rice seed coat, colored rice can be distinguished into black rice, purple rice, red rice, yellow rice, etc. [1]. Cyanidin-3-O-glucoside (C3G) and peonidin-3-O-glucoside (P3G) are the primary anthocyanidins found in the seed coat of black rice, whereas red rice contains abundant protocatechuic acid (PA) and oligomers of flavan-3-ols [2]. Red rice is a characteristic natural feature of wild rice and the precursor of cultivated rice. Due to functional mutations at the *Rc* locus, the red seed coat is lost in most cultivated rice varieties. Black rice originates from the functional mutation of the *OsB2* locus. *OsB2* is the *bHLH* gene that regulates the synthesis of anthocyanidins in rice. Modification of the promoter region of *OsB2015* results in a notable increase in the level of anthocyanidin expression in both seeds and leaves [3]. According to related studies, red rice contains significantly more His, Lys, Tyr, and Tau than other colors of rice, and black rice contains significantly more Arg, Asn, Glu, Met, Orn, and Try than other colors of rice, which proves that red and black rice have greater nutritional value [4].

Purple rice contains anthocyanins, phenolics, glutenin, γ-aminobutyric acid, and other beneficial components that have diverse physiological effects such as antioxidant, blood lipid-regulating, and blood glucose level-controlling properties [5,6,7]. According to related research, the presence of anthocyanidins and phenolic compounds in black rice can increase the expression of genes related to adipogenesis and fatty acid oxidation in the liver by activating the *AMPK* and *PPAR* signaling pathways, thereby inhibiting obesity [8]. In addition, black rice contains a high amount of C3Gg. Administering black rice to rats for a duration of 8 weeks has the potential to diminish blood sugar levels and alleviate oxidative stress and inflammation. Additionally, black rice can inhibit the transforming growth factor β1/Smad2/3 pathway, leading to the restoration of diabetic nephropathy and renal pathological alterations in rats. Thus, black rice can be used as a nutrient for humans to prevent diabetic nephropathy [9]. Red rice plants are rich in anthocyanidins, tocopherols, tocotrienols, iron, zinc, and vitamins [10,11,12]. Red rice has cholesterol-lowering [13] and anticancer properties [14]. Related research shows that the main anthocyanidin in red rice is paeoniflorin, which has good antioxidant and tyrosinase inhibitory activities and is used in the fields of health food, medicine, and beauty [15]. In addition to these flavonoids and phenolic compounds, the biological activities of many oxidoreductase enzymes, including catalase (CAT), peroxidase (POD), and polyphenol oxidase (PPO), are closely related to antioxidant activity. Among these enzymes, CAT can prevent the production of hydroxyl radicals and performs a functional role in protecting the antioxidant enzyme system. PPO converts polyphenols into quinones, which are more powerful antioxidants that prevent cell oxidation and aging. These bioactive substances affect the total antioxidant capacity of the organism or biological tissue.

Metabolomics has unique advantages in the qualitative and quantitative analysis of small molecule metabolic components. Several researchers used metabolomics methods for a comprehensive analysis of the metabolites of 16 rice varieties, and a total of 110 metabolites were detected. The analysis revealed that black rice contains metabolites derived from the terpenoid and phenylpropanoid biosynthesis pathways at elevated levels, whereas red rice exclusively contains high-level metabolites from the phenylpropanoid biosynthesis pathway. On the other hand, white rice is characterized by the presence of secondary metabolites at lower levels. This is the first evidence of the relationships and metabolic differences in terpenoid content between white rice and colored rice [9]. Korean scholars have used metabonomic methods to quantitatively analyze the antioxidant, total flavonoid, total phenol, anthocyanidin contents, and other indices of 29 rice varieties. The authors found that the antioxidant activity of these rice samples and the contents of total phenolic compounds (TPCs), total flavonoid compounds (TFCs), and total antioxidant compounds (TACs) were significantly different, which confirmed the close relationship between phenolic components and antioxidant activity [16].

This study focused on the colored rice varieties YZ6H (black rice), YJN818 (golden rice), HN (red rice), and YCN1H (white rice). We used untargeted metabolomics techniques to analyze the metabolites of different colored rice grains and to identify the relevant metabolites quantitatively. In this study, we used analysis of variance (ANOVA), correlation analysis, and significance analysis to explore the compositional differences and associations between physiological and biochemical indices and metabolites in different colored rice grains to examine the link between rice metabolites and overall antioxidant capacity, to determine the enrichment level of total antioxidants in different colored rice grains, and to identify the key substances that affect the antioxidant capacity of colored rice grains. This study provides a theoretical basis for breeding colored rice plants with high antioxidant capacity.

## 2. Materials and Methods

### 2.1. Botanical Substances and Cultivation Circumstances

The experimental materials Yangjinnuo 818 (YJN818), Hongnuo (HN), Yangchannuo 1 hao (YCN1H), and Yangzi 6 hao (YZ6H) were grown by the Agricultural College of Yangzhou University. The trial samples were sown at Shatou Base in Guangling District, Yangzhou City, Jiangsu Province. The rice planting date is 25 May 2022. After 20 days of growth, the seedlings were transplanted at a row spacing of 12 × 30 cm, with 4 seedlings planted in each hole. Three replicates were placed in the field, with each plot measuring 30 square meters (5 × 6 m). Control of pests, diseases, and weeds was conducted according to conventional high-yield rice cultivation requirements.

### 2.2. Sample Collection

After the rice plants were ripe and harvested, the rice grains of YZ6H, YJN818, HN, and YCN1H were ground into brown rice, and each variety was sampled three times (a single replicate was a mixture of 10 panicles of grains). For every variety of rice seed, three biological replicates were collected. Following the sampling process, the samples were promptly transferred into a liquid nitrogen container, transported to the laboratory, and preserved in a −80 °C ultracold freezer for subsequent use. The sample numbers were YZ6H_ 1, YZ6H_ 2, YZ6H_ 3, YJN818_ 1, YJN818_ 2, YJN818_ 3, HN_ 1, HN_ 2, HN_ 3, YCN1H_ 1, YCN1H_ 2, and YCN1H_ 3.

### 2.3. Measurement of Physiological and Biochemical Indices

Physiological and biochemical data from rice grain samples were obtained by measuring catalase (CAT) activity [17], polyphenol oxidase (PPO) activity [18], phenylalanine ammonia-lyase (PAL) activity [19], peroxidase (POD) activity [20], oligomeric proanthocyanidin (OPC) content [21], and total protein (TP) content [22]. The measurements were conducted using the 2,2′-azino-bis(3-ethylbenzothiazoline-6-sulfonic acid) (ABTS) method [23], the 2,2-diphenyl-1-picrylhydrazyl (DPPH) method [24]), and the ferric ion reducing antioxidant power (FRAP) method [25,26]. The assay kits used for these measurements were provided by Suzhou Michy Biomedical Technology Co., Ltd. (Suzhou, China). Specific indices were extracted and measured as follows:

#### Sample Preparation

CAT, POD, SOD, PPO, PAL, ABTS, DPPH, FRAP: Approximately 0.1 g of sample was weighed out, and 1 mL of extraction solution was added. The mixture was shaken well in an ice bath and centrifuged at 12,000 rpm and 4 °C for 10 min. The supernatant was collected and placed on ice until analysis.

TP: The sample was dried, pulverized, and passed through a 40-mesh sieve. Approximately 0.02 g of the sample was then added to 1 mL of extraction solution and extracted by shaking at 60 °C for 2 h. After centrifugation at 10,000× *g* and 25 °C for 10 min, the supernatant was collected.

OPC: Approximately 0.05 g of dried sample was combined with 1 mL of extraction solution, homogenized, and extracted by shaking at 60 °C for 2 h. After centrifugation at 10,000× *g* and 25 °C for 10 min, the supernatant was collected.

Flavonoid: The sample was dried, pulverized, and passed through a 40-mesh sieve. Then, approximately 0.05 g of sample was weighed, 1 mL of 60% ethanol was added, and the extraction was carried out by shaking at 60 °C for 2 h. The mixture was centrifuged at 10,000× *g* and 25 °C for 10 min, and the supernatant was collected.

The Microplate Reader was preheated for more than 30 min, and the wavelength settings of the Microplate Reader were as follows: CAT (240 nm), POD (470 nm), SOD (450 nm), PPO (525 nm), PAL (290 nm), TP (765 nm), OPC (500 nm), Flavonoid (510 nm), ABTS (734 nm), DPPH (515 nm), and FRAP (593 nm).

### 2.4. Extraction, Identification, and Analysis of Metabolites

Xiong et al. provided a reference for the extraction, detection, and analysis of metabolites [27]. A sample of rice seeds weighing 50 mg was placed in a centrifuge tube with a capacity of 2 mL. Subsequently, a grinding bead with a diameter of 6 mm was introduced. To extract metabolites, 400 μL of extraction solution (4:1 methanol/(*v*/*v*)) with an internal standard (L-2-chlorophenylalanine) concentration of 0.02 mg mL^−1^ was added. The sample was crushed in a frozen tissue pulverizer for 6 min at −10 °C and a frequency of 50 Hz. Subsequently, the proteins were extracted through cryosonication for 30 min at 5 °C with a frequency of 40 kHz. The samples were permitted to remain at a temperature of −20 °C for a duration of 30 min and then subjected to centrifugation for 15 min at a temperature of 4 °C and a force of 13,000× *g*. Subsequently, the resulting supernatant was transferred into an injection vial using an internal cannula for further analysis.

The preprocessed matrix file was examined for differences. The R software package ropls (version 1.6.2) was used for conducting principal component analysis (PCA) and orthogonal least squares discriminant analysis (OPLS−DA). The model’s stability can be evaluated by using 7 cycles of interactive validation. Furthermore, Student’s *t*-test and analysis of variation (ANOVA) were also conducted. Differentially abundant metabolites (DAMs) were chosen by considering the variable importance in the projection (VIP) values derived from the OPLS−DA model and the *p* values from Student’s *t*-test. Metabolites with VIP > 1 and *p* < 0.05 were identified as DAMs. A total of 825 DAMs were screened. The KEGG database (https://www.kegg.jp/kegg/pathway.html (accessed on 24 November 2022) was utilized to annotate the DAMs and determine their associated pathways.

### 2.5. Quantitative Detection of Flavonoids and Phenolic Substances

The quantification of flavonoid metabolites followed the methods of Xiong et al. (2023) [28]. Precise measurements were taken using an analytical balance for the flavonoids and phenolic compounds, and detailed information about the standards can be found in Supplementary Materials Table S1. To achieve a stock solution concentration of 1 mg mL^−1^, the solution was mixed with methanol until fully dissolved. Various gradient working solutions were prepared by diluting the stock solution to concentrations of 200 ng mL^−1^, 80 ng mL^−1^, 32 ng mL^−1^, 12.80 ng mL^−1^, 5.12 ng mL^−1^, 2.05 ng mL^−1^, 0.82 ng mL^−1^, 0.33 ng mL^−1^, 0.13 ng mL^−1^, 0.05 ng mL^−1^, and 0.02 ng mL^−1^. The UPLC–ESI–MS/MS analysis method was used in this study to perform quantitative detection of flavonoid and phenolic compounds.

The chromatographic conditions were as follows:

Injection volume: 5 μL. Flow rate: 0.35 mL min^−1^.

Mobile phase: Phase A: 0.1% formic acid—aqueous solution; Phase B: acetonitrile. The gradient elution procedure was as follows: 0 min: A/B (95:5, *v*/*v*); 0.8 min: A/B (95:5, *v*/*v*); 3 min: A/B (75:25, *v*/*v*); 12 min: A/B (56.2:43.8, *v*/*v*); 13 min: A/B (1:99, *v*/*v*); 14.4 min: A/B (1:99, *v*/*v*); 14.41 min: A/B (99:5, *v*/*v*); 15 min: A/B (95:5, *v*/*v*).

The mass spectrometry conditions were as follows:

Positive Ionization Mode: CUR: 35 psi; EP: 10; IS: 5500; CXP: 10; TEM: 500 °C; Gas1: 60 psi; Gas2: 50 psi. Negative Ion Mode: CUR: 35 psi; EP: −10; IS: −4500; CXP: −20; TEM: 500 °C; Gas1: 60 psi; Gas2: 50 psi. Column temperature: 40 °C.

### 2.6. Data Analysis of Flavonoids and Phenolic Substances

The default parameters in the SCIEX OS-MQ software (Redwood, CA, USA) were used to automatically identify and integrate each MRM transition with the assistance of manual inspection. For each index, the samples exhibited excellent peak shapes and high separation in terms of the total ion current chromatogram (TIC) (Supplementary Materials Figure S1A), as did the negative extracted ion chromatograms (XICs) (Supplementary Materials Figure S1B) and positive XICs (Supplementary Materials Figure S1C) of the substances. The horizontal coordinate of the XIC represents the retention time (RT) of the metabolite assay, while the vertical coordinate represents the ion intensity (cps) of the ion assay. The mass spectral peaks detected for each metabolite in the various samples were manually adjusted to ensure qualitative and quantitative accuracy. This adjustment was based on information regarding the retention time and shape of the peak, while the area of each chromatographic peak indicated the relative content of the corresponding metabolite. The standard curves of different metabolites were plotted according to the corresponding quantitative mass spectral data of the standards at different concentrations using the concentration of the standards as the horizontal coordinate and the peak areas of the mass spectral peaks as the vertical coordinate. The linearity of the standard curves for various metabolites was excellent, with an R^2^ value greater than 0.99 (Supplementary Materials Table S2). Mass spectrometry detection of various QC samples (Supplementary Materials Figure S1) revealed that the TICs of the metabolite assay exhibited significant curve overlap, indicating consistent signal stability across different time intervals for both the mass spectrometry and the liquid chromatography systems. The relative standard deviation (RSD) for each metabolite was less than 15.71% (Supplementary Materials Table S2), demonstrating the stability and reliability of this method and the analytical system, which made them suitable for quantitative sample detection. Supplementary Materials Table S2 displays the RT, detection limit, and quantitation limit for each standard. To obtain concentration information for each sample metabolite, a regression equation fitted from the standard curve was used to determine the peak area.

### 2.7. Physiological and Biochemical Data Analysis

Physiological and biochemical data were arranged and plotted using Adobe Illustrator CS6 and WPS2021. Analysis of variance (ANOVA), correlation analysis, and significance analysis (Tukey’s test) were performed using SPSS 18.0 software. Related tasks, such as multivariate statistical analysis, were performed using Version 1.6.20. KEGG compound classification and functional pathway enrichment analyses were also performed using KEGG Pathway (accessed on 1 May 2017).

## 3. Results

### 3.1. Analysis of Physiological and Biochemical Indices

**Figure 1:**
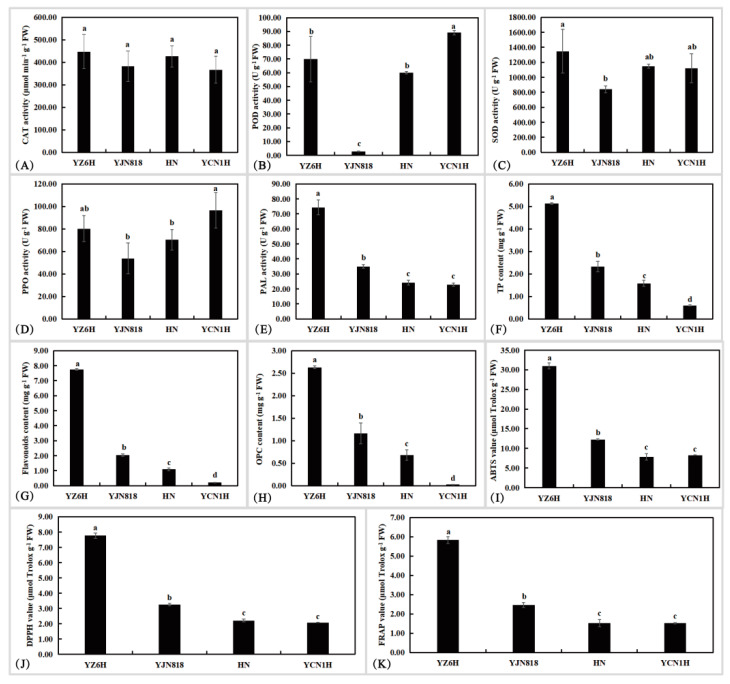
Analysis of differences in physiological and biochemical indices of colored rice grains: (**A**) CAT activity; (**B**) POD activity; (**C**) SOD activity; (**D**) PPO activity; (**E**) PAL activity; (**F**) TP content; (**G**) flavonoid content; (**H**) OPC content; (**I**) ABTS (2,2′-azino-bis(3-ethylbenzothiazoline-6-sulfonic acid) value; (**J**) DPPH (2,2-diphenyl-1-picrylhydrazyl) value; (**K**) FRAP (ferric ion reducing antioxidant power) value. Different lowercase letters indicate significance at the *p* = 0.05 level.

In this research, physiological and biochemical markers such as CAT activity, POD activity, SOD activity, PPO activity, PAL activity, TP content, flavonoid content, OPC content, ABTS radical scavenging capacity, DPPH radical scavenging capacity, and FRAP were detected in grain samples (Figure 1). YJN818 had the lowest POD activity, which was 0.04, 0.047, and 0.032 times greater than that of YZ6H, HN, and YCN1H, respectively. YZ6H exhibited the highest PAL activity, which was 2.13, 3.08, and 3.25 times greater than that of YJN818, HN, and YCN1H, respectively. The contents of TP, flavonoids, and OPC were significantly different among the four different colored grain samples. The TP content of YZ6H was 2.20, 3.25, and 8.55 times greater than that of YJN818, HN, and YCN1H, respectively. The flavonoid content of YZ6H was 3.80, 7.10, and 35.24 times greater than that of YJN818, HN, and YCN1H, respectively. The OPC content of YZ6H was 2.26, 3.87, and 104.10 times greater than that of YJN818, HN, and YCN1H, respectively (Figure 1). YZ6H exhibited the greatest overall antioxidant capacity, as determined by the ABTS, DPPH, and FRAP techniques, with YJN818 and YCN1H ranking next, suggesting that darker rice grains possess higher levels of antioxidant metabolic compounds.

Significant variations in PAL activity, total antioxidant capacity, TP content, flavonoid content, and OPC content were detected among colored rice grains, such as YZ6H, and white rice grains, such as YCN1H, as evidenced by comprehensive physiological and biochemical data. Overall, significant variations were observed in the antioxidant enzyme activity, total antioxidant capacity, TP content, flavonoid content, and total phosphorus content among the YZ6H, YJN818, HN, and YCN1H grains. In plants, PAL is a key enzyme in the phenylalanine metabolic pathway that promotes the synthesis of flavonoids and phenolics. As shown in Figure 1E,F,I–K, the content of phenylalanine deaminase indirectly reflects the content of flavonoids and anthocyanins as well as the overall antioxidant property of YZ6H, which verifies the reliability of the results of the study.

### 3.2. Multiple Statistical Analysis

**Figure 2:**
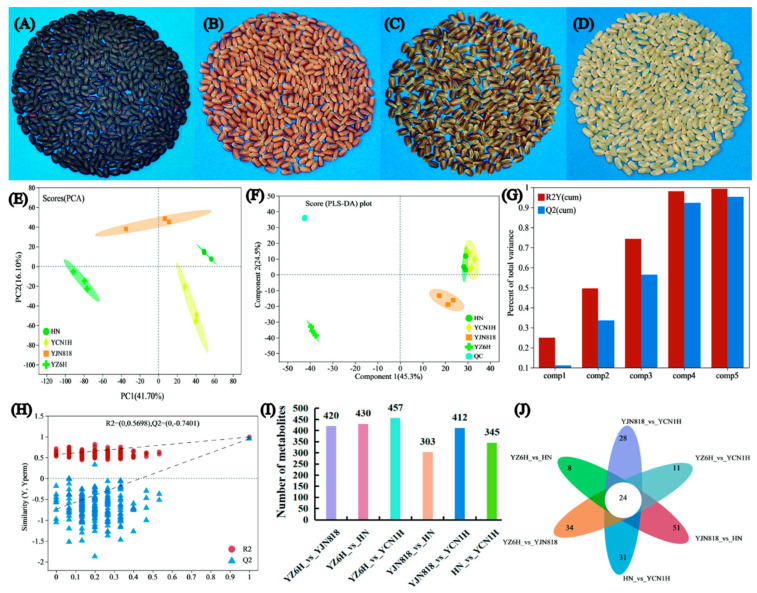
Overview of grain metabolite information: (**A**) YZ6H grain sample, (**B**) HN grain sample, (**C**) YJN818 grain sample, (**D**) YCN1H grain sample, (**E**) PCA score, (**F**) PLS-DA permutation test, (**G**) Overview of the PLS-DA model, (**H**) PLS-DA permutation test, (**I**) Comparison of differentially abundant metabolites between groups, and (**J**) Venn diagram of the groups.

By comparing the grain morphology data of YZ6H, YJN818, HN, and YCN1H, it was observed that there were notable variations in grain color among the four rice cultivars (lines) (Figure 2A–D). We compared the main metabolic components of the different samples and obtained a preliminary understanding of the intragroup differences in flavonoid and antioxidant enzyme activities among these four different varieties of rice. PCA is a highly efficient technique for detecting primary compounds in high-throughput spectra. The PCA score plot revealed that principal component (PC) 1 accounted for 41.7% of the variance, while PC 2 accounted for 16.1% of the variance. Together, PCs 1 and 2 accounted for 57.8% of the variance in the data (Figure 2E). The main characteristic information of the rice grain samples can be reflected by the first two PCs, and there are notable variations in the PCs among the grains of four distinct colors. Further, the PLS-DA of the data showed that Component 1 could explain 45.3% of the variation in the data, while Component 2 could explain 42.5% of the variation in the data (Figure 2F). This indicated a significant difference in the PCs between YZ6H and YJN818, while there was a certain degree of component crossover between YCN1H and HN, which was relatively consistent with the PCA results. The PLS-DA modeling and prediction abilities can be evaluated using R^2^Y and Q^2^, respectively. Figure 1G illustrates that as the combined R^2^Y and Q^2^ increase, the model becomes more dependable and consistent. The upward trend of the regression line, along with decreases in R^2^ and Q^2^, indicated that the model did not exhibit overfitting (Figure 2H).

We analyzed the differential abundance of metabolites in the different comparison groups, with a focus on flavonoids and phenolic compounds. YZ6H and YJN818 had 50 differentially abundant flavonoids, or 16.50%. YZ6H and HN had 51 differentially abundant flavonoids, or 16.14% (Supplementary Materials Figure S2B; Supplementary Materials Table S4); YZ6H and YCN1H had 53 differentially abundant flavonoids, or 16.16% (Supplementary Materials Figure S2C; Supplementary Materials Table S5); YJN818 and HN had 42 differentially abundant flavonoids, or 19.35% (Supplementary Materials Figure S2D; Supplementary Materials Table S6); YJN818 and YCN1H had 38 differentially abundant flavonoids, or 12.46% (Supplementary Materials Figure S2E; Supplementary Materials Table S7); and HN and YCN1H had 29 differentially abundant flavonoids, or 11.28% (Supplementary Materials Figure S2F; Supplementary Materials Table S8). These data suggest that flavonoids are one of the main components influencing the differences between groups (Figure 2I).

Furthermore, we generated volcano plots of the distinct metabolites of the various comparison groups (Supplementary Materials Figure S3). Venn diagram analysis of the distinct metabolites in the various comparison groups revealed that each group had unique metabolites, suggesting variations in metabolic composition among the different groups (Figure 2J).

### 3.3. KEGG Analysis

Kyoto Encyclopedia of Genes and Genomes (KEGG) enrichment analysis revealed significant pathway involvement of DAMs between the YZ6H and YJN818 comparison groups. Table 1 shows the pathways that were significantly different (*p* < 0.05): anthocyanin biosynthesis, flavone and flavonol biosynthesis, and flavonoid biosynthesis. The direct interactions between the YZN6H and HN comparison groups were associated with various metabolic pathways, such as the synthesis of flavones, flavonols, and flavonoids (*p* < 0.05). The YZ6H and YCN1H comparison groups were shown to be involved in several metabolic pathways, such as the biosynthesis of phenylalanine, tyrosine, tryptophan, anthocyanins, flavones, flavonols, and flavonoids (*p* < 0.05). The direct signals exchanged between the YJN818 and HN comparison groups were associated with various metabolic pathways, such as the synthesis of flavonoids as well as the metabolism of alanine, aspartate, and glutamate (*p* < 0.05). The YJN818 and YCN1H comparison groups were shown to be involved in various metabolic pathways, such as anthocyanin production; biosynthesis of phenylalanine, tyrosine, and tryptophan; phenylpropanoid production; flavone and flavonol synthesis; and flavonoid production (*p* < 0.05). The direct interactions between the HN and YCN1H comparison groups were shown to be associated with several metabolic pathways, such as the synthesis of tyrosine and tryptophan, the production of anthocyanins, the biosynthesis of flavones and flavonols, the formation of phenylpropanoids, and the synthesis of flavonoids (*p* < 0.05).

**Figure 3:**
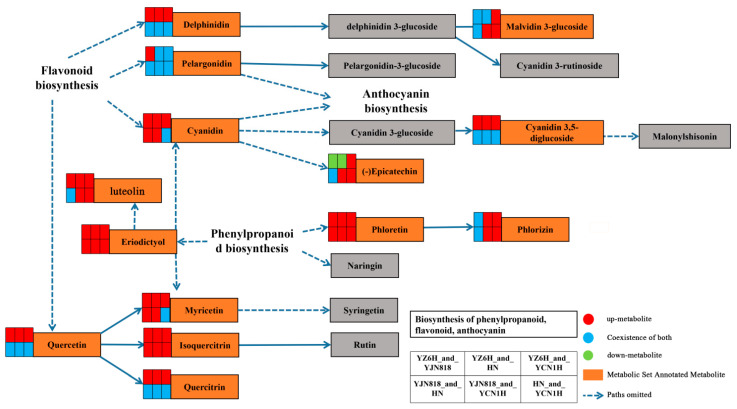
Schematic diagram of the phenylpropanoid, flavonoid, and anthocyanin synthesis pathways. Orange boxes represent key differentially abundant metabolites; red squares represent higher-abundance metabolites; green squares represent lower-abundance metabolites; blue represents the coexistence of both; and arrows represent synthesis paths.

The key substances involved in the metabolic pathway of phenylpropanoid, flavonoid, and anthocyanin synthesis were found to be eriodictyol, luteolin, phloretin, and phlorizin from the synthetic pathway of phenylpropanoid, flavonoid, and anthocyanin, respectively, with delphinidin, pelargonidin, cyanidin, and quercetin being the key substances in pelargonidin and cyanidin. The key substances involved in the metabolic pathway of flavonoid biosynthesis were delphinidin, pelargonidin, cyanidin, and quercetin, of which pelargonidin and cyanidin continue to be involved in the anthocyanin biosynthesis metabolic pathway (Figure 3).

### 3.4. Quantitative Analysis of Flavonoids and Phenolic Compounds

A total of 129 flavonoids and phenolic compounds were detected in this study. Among them, 78 flavonoids and phenolic compounds were present at low concentrations or were not present, and their metabolites were not present. Hence, this research quantitatively examined 51 flavonoids and phenolic compounds in four distinctly colored grain types (Table 2).

All four rice grain types, which had varying colors, were shown to contain high levels of 4-hydroxycinnamic acid, cyanidin-3-O-rutinoside chloride (C3R), ferulic acid, isoorientin, isorhamnetin-3-O-glucoside, pelargonidin-3-glucoside, protocatechuic acid, quercetin-3-galactoside, rutin, salicylic acid, sinapic acid, taxifolin, and vanillic acid. YZ6H had high levels of eriodictyol, gallic acid, gentisic acid, hesperidin, isoorientin, isorhamnetin, isorhamnetin-3-O-glucoside, methyl gallate, morin, orientin, protocatechuic acid, prunin, quercetin-3-galactoside, quercetin, rutin, salicylic acid, syringic acid, taxifolin, trans-piceid, and vitexin, while YCN1H had the lowest levels. YZ6H exhibited significantly greater levels of 32 flavonoid metabolites, according to the quantitative analysis of flavonoids and phenolic compounds. Among them, protocatechuic acid (PA), cyanidin-3-O-rutinoside chloride (C3R), and vanillic acid were the three most common flavonoids and phenols in YZ6H, together accounting for 67.16% of the total content. The PA content in YZ6H was 20.41, 116.13, and 494.95 times greater than that in YJN818, HN, and YCN1H, respectively; the C3R content in YZ6H was 686.14 and 2781.33 times greater than that in HN; and the vanillic acid content in YZ6H was 57.08, 32.84 and 36.10 times greater than that in YJN818, HN and YCN1H, respectively. YCN1H had higher amounts of aromadendrin and ferulic acid, while YZ6H had the lowest levels of these compounds. YZ6H contained the metabolites chlorogenic acid, myricetin-3-galactoside, naringin, pelargonidin-3-glucoside, phloretin, phlorizin, trilobatin, and trans-3,3′,4′,5,5′,7-hexahydroxyflavanone, whereas the other samples (Table 2) did not contain these metabolites.

### 3.5. Evaluation of the Relationships between Physiological and Biochemical Indices and Flavonoid Metabolites through Correlation Analysis

To investigate the relationships between flavonoid metabolites and physiological and biochemical indices, we performed correlation analysis between 51 flavonoid metabolites and the activity of the physiological and biochemical indices CAT, POD, SOD, PPO, and PAL; TP, flavonoid, and OPC contents; ABTS and DPPH radical scavenging activities; and FRAP.

The study revealed 36 metabolites, such as phlorizin, trans-3,3′,4′,5,5′,7-hexahydroxyflavanone, and trilobatin, that exhibited strong and significant correlations (*p* < 0.01) with PAL activity; flavonoid, TP, and OPC content; ABTS and DPPH radical scavenging activity; and FRAP (Table 3). Combined with the results of the quantitative analysis of flavonoids and phenolics, we preliminarily concluded that the 36 metabolites might be the main substances affecting the color of the rice grains. The higher the content of these metabolites was, the darker the color of the rice grains.

### 3.6. Receiver Operating Characteristic (ROC) Analysis

**Figure 4:**
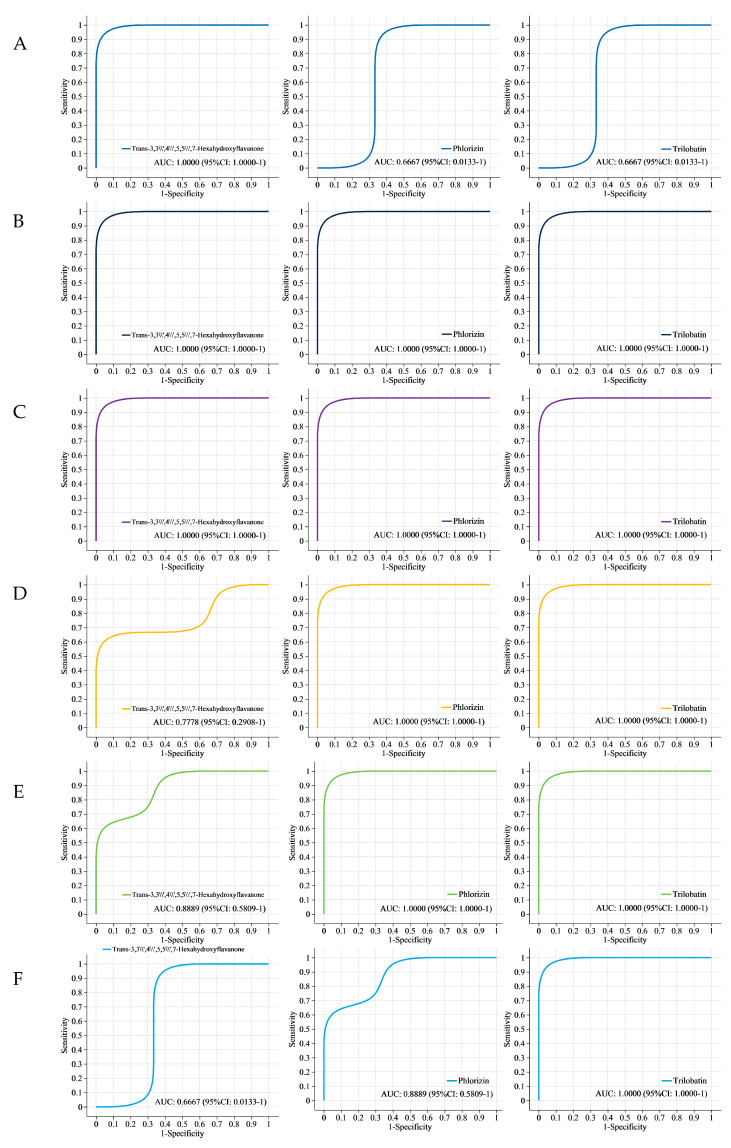
ROC analysis: (**A**) ROC analysis of trans-3,3′,4′,5,5′,7-hexahydroxyflavanone, phlorizin, and trilobatin between YZ6H and YJN818; (**B**) ROC analysis of trans-3,3′,4′,5,5′,7-hexahydroxyflavanone, phlorizin, and trilobatin between YZ6H and HN; (**C**) ROC analysis of trans-3,3′,4′,5,5′,7-hexahydroxyflavanone, phlorizin, and trilobatin between YZ6H and YCN1H; (**D**) ROC analysis of trans-3,3′,4′,5,5′,7-hexahydroxyflavanone, phlorizin, and trilobatin between YJN818 and HN; (**E**) ROC analysis of trans-3,3′,4′,5,7′,7-hexahydroxyflavanone, phlorizin, and trilobatin between YJN818 and YCN1H; (**F**) ROC analysis of trans-3,3′,4′,5′,7-hexahydroxyflavanone, phlorizin, and trilobatin in HN and YCN1H.

Figure 4A shows that the area under the curve (AUC) values for trans-3,3′,4′,5,5′,7-hexahydroxyflavanone, phlorizin, and trilobatin between YZ6H and YJN818 were 1, 0.6667, and 0.6667, respectively. This suggests a notable disparity in the content of trans-3,3′,4′,5,5′,7-hexahydroxyflavanone present in the YZ6H and YJN818 grains. The AUC for trans-3,3′,4′,5,5′,7-hexahydroxyflavanone, phlorizin, and trilobatin between YZ6H and HN was 1 for all three compounds (Figure 4B). This indicated that a large amount of flavonoids accumulated in the YZ6H grains, and there was a significant difference in the total antioxidant capacity between the YZ6H and HN grains. The AUC values for trans-3,3′,4′,5,5′,7-hexahydroxyflavanone, phlorizin, and trilobatin between YZ6H and YCN1H were all equal to 1, as shown in Figure 4C. Moreover, as shown in Figure 4D, the AUC values for YJN818 and HN were 0.7778, 1, and 1 for trans-3,3′,4′,5,5′,7-hexahydroxyflavanone, phlorizin, and trilobatin, respectively. Figure 4E shows that the AUC values for trans-3,3′,4′,5,5′,7-hexahydroxyflavanone, phlorizin, and trilobatin were 0.8889, 1, and 1, respectively, in the comparison between YJN818 and YCN1H. Figure 4F shows that the AUC values for trans-3,3′,4′,5,5′,7-hexahydroxyflavanone, phlorizin, and trilobatin were 0.6667, 0.8889, and 1, respectively, when comparing HN and YCN1H. The above results indicate that a large amount of flavonoids accumulate in HN grains and that the difference in total antioxidant capacity between HN and YCN1H grains is significant.

## 4. Discussion

The nutritional value of colored rice is greater than that of white rice, as colored rice contains essential amino acids, vitamins, phenolic compounds, flavonoids, and other beneficial substances [29]. In this study, significant differences were observed in the total antioxidant power, TP content, flavonoid content, and OPC content among the four different colors of rice grains. YZ6H exhibited the highest values, while YCN1H had the lowest values (Figure 1). According to previous research, black rice bran has been found to possess a DPPH radical scavenging capacity that is 5.4 times greater than that of red rice bran and 16 times greater than that of white rice bran [30]. Black rice has higher levels of total phenols, flavonoids, and anthocyanins than red and white rice, indicating greater antioxidant activity [31]. Darker varieties exhibit greater amounts of phenolic flavonoids and greater antioxidant activity than lighter varieties, as observed from the standpoint of rice seed coat color [32]. To determine the relevant synthesis pathways that impact the variations in the antioxidant capability of rice grains, we performed a KEGG analysis, which revealed that the primary metabolic pathways distinguishing the different comparison groups were phenylalanine, tyrosine, and tryptophan biosynthesis; anthocyanin biosynthesis; flavone and flavonol biosynthesis; and flavonoid biosynthesis. Taken together, these findings indicate that phenolic and flavonoid metabolites were significantly enriched in all six comparison groups and were the keys to influencing the antioxidant properties of these four different colored rice seeds (Table 1).

We found from the analysis of the compound statistical data that the proportion of flavonoids in the three comparison groups related to YZ6H was greater than 16%, and the proportion of flavonoids in the three comparison groups relative to that in YCN1H was greater than 11% (Supplementary Materials Figure S2), indicating a significant difference in flavonoid content between colored rice and white rice. The DAMs between colored and white rice were significantly enriched mainly in isoflavones, flavonoids, flavonols, and flavonoid biosynthetic pathways. The analysis of gene expression patterns revealed 227 differentially expressed genes (DEGs) between colored and white rice. Of these DEGs, 173 were upregulated and were found to be enriched in the pathway responsible for the production of flavonoids [33]. Furthermore, these findings support the perspective that the metabolites of the variously pigmented rice comparison groups are predominantly concentrated in the pathway responsible for flavonoid biosynthesis [34]. Anthocyanins, which are flavonoids, are plentiful natural antioxidants found in the human diet. They can effectively eliminate free radicals, thereby preventing numerous diseases [35]. Therefore, exploring colored rice enriched in antioxidant substances can increase the natural antioxidant intake required for human dietary structure and health benefits.

To investigate the primary components that impact the antioxidant properties of rice kernels, we employed mass spectrometry techniques to quantitatively examine the distinct compounds found in these four varieties of rice grains. Through this analysis, we successfully identified 51 unique metabolites belonging to the flavonoid and phenol families. Among them, protocatechuic acid (PA), C3R, and vanillic acid were the three most common flavonoids and phenols in YZ6H, which together accounted for 67.16% of the total content. The PA content of YZ6H was 20.41, 116.13, and 494.95 times greater than that of YJN818, HN, and YCN1H, respectively; the C3R content of YZ6H was 686.14 and 2781.33 times greater than that of YJN818 and YCN1H, respectively, and C3R was not detected in HN; and the vanillic acid content of YZ6H was 57.08, 32.84 and 36.10 times greater than that of YJN818, HN and YCN1H, respectively (Table 2). PA and vanillic acid, identified as antioxidants and tyrosinase inhibitors [36], are the primary phenolic compounds found in black rice extracts. Thai researchers have proven that PA, found in purple rice bran, can prevent the development of diethylnitrosamine-induced liver precancerous lesions in rats, demonstrating its anticancer effects [37]. Vanillic acid can exert neuroprotective effects under oxidative stress and help treat Alzheimer’s disease [38]. The most abundant substance in YJN818 and HN was found to be procyanidin B3 (Table 3), which scavenges DPPH free radicals and hydroxides, strongly inhibits α-glucosidase, delays glucose uptake in type 2 diabetes patients, and is a natural agent for the treatment of patients with diabetes [39]. Related studies have confirmed that red rice color is deepened through the oxidation of intramolecular bonds in procyanidin [40]. Notably, we detected a large amount of ferulic acid in YCN1H, while YZ6H had the lowest ferulic acid content, and there was a significant difference in ferulic acid content among these four varieties (Table 2). Previous research has shown that white rice contains elevated amounts of ferric acid and 4-hydroxycinnamic acid, with ferric acid being the predominant phenolic acid found in whole grains. Additional verification is required to ascertain whether the metabolites of ferric acid can function as indices of antioxidant efficacy. The contents of catenin and epicatechin in red rice are high, and quercetin and catenin have peak antioxidant capacities in black and red rice, respectively [41]. YJN818 and HN had higher levels of catenin than the other samples, and only epicatechin was observed in YJN818 (Table 2), which aligns with previous findings. The anthocyanins found in black rice primarily consist of C3G, with a lesser amount of pelargonidin-3-glucoside and C3R [42]. Combining the physiological and biochemical data (Figure 1) and the individual contents of 51 flavonoids and phenolics in these four different colors of rice grains (Table 2), we found that the total antioxidant capacity of the four varieties was consistent with the distribution trends of the TP and flavonoid contents, and the differences were significant. Similarly, the flavonoid and phenolic contents of YZ6H were 1.25, 3.02, and 9.30 times greater than those of YJN818, HN, and YCN1H, respectively. These differences were also significant, thus proving that YZ6H, a type of black rice, has more antioxidant substances than other colors of rice, such as red and white rice. This difference may provide a new direction for increasing the consumption of antioxidants in the human diet. A schematic diagram of the study revealed that pelargonidin and cyanidin are crucial components of the flavonoid biosynthesis pathway. These substances undergo transformation into pelargonidin-3-glucoside and C3G (Figure 3). We detected C3R and pelargonidin-3-glucoside metabolites in YZ6H (Table 3), which may be unique to black rice varieties.

Through correlation analysis with physiological and biochemical indices, this study revealed highly significant relationships between these substances and total antioxidant capacity, TP content, flavonoid content, and OPC content (Table 3), and these metabolites can be used as candidate biomarkers for antioxidant activity. ROC analysis can determine the similarity between different sampling groups to verify the significance of differences between them, and supervised learning methods can be used to perform linear discrimination and classification modeling on the sampling groups to detect the main metabolites involved in group differentiation [43]. To confirm the dependability of the findings, we employed ROC curve analysis to assess three metabolites: phorizin, trans-3,3′,4′,5,5′,7-hexahydroxyflavanone, and trilobatin (Figure 4). In the six comparison groups, the AUC values of these three metabolites were greater than 0.6667, indicating good diagnostic efficacy. The antioxidant properties of YZ6H were significantly different from those of HN and YCN1H. The AUC values of the three key metabolites, phospholipin, trans-3,3′,4′,5,5′,7-hexahydroxyflavanone, and trilobatin, were all 1 in both the YZ6H and HN comparison groups and the YZ6H and YCN1H comparison group. These findings indicate that these metabolites have excellent diagnostic efficacy as antioxidant biomarkers. A schematic diagram of the pathways associated with the synthesis of phenylpropanoids, flavonoids, and anthocyanins revealed that phlorizin is a key metabolite generated through the transformation of phloretin to the phenylpropanoid biosynthesis pathway, and phloretin was upregulated in all six comparison groups (Figure 3). Hence, these three compounds could play a crucial role in influencing the antioxidant properties of rice kernels. This study focused on these three metabolic markers during the selection and breeding of rice varieties, and this information could help to determine whether a given variety contains abundant antioxidant substances.

## 5. Conclusions

Compared with white rice grains (YCN1H), colored rice grains (YZ6H, YJN818, and HN) exhibited higher antioxidant enzyme activity, total antioxidant capacity, TP content, flavonoid content, and OPC content. An analysis of the color of the rice seed coat revealed that the darker the color was, the greater the contents of anthocyanins, flavonoids, and other compounds in the rice grains were, resulting in more potent overall antioxidant capacity. This study identified 32 metabolites that affect the antioxidant activity of YZ6H from 51 flavonoids and phenolic metabolites shared by six comparative groups of four different colored rice grains. Chlorogenic acid, myricetin-3-galactoside, naringin, pelargonidin-3-glucoside, phospholipin, porphyrin, trans-3,3′,4′,5,5′,7-hexahydroxyflavanone, and trilobatin are the distinctive compounds found in black rice. Correlation analysis between physiological and biochemical indices and these metabolites revealed significant differences. Moreover, when the ROC curves were combined, the AUC values of trans-3,3′,4′,5,5′,7-hexahydroxyflavanone, philorizin, and trilobatin were all 1 in both the YZ6H and HN comparison and the YZ6H and YCN1H comparison. Hence, the investigation revealed that the metabolites trans-3,3′,4′,5,5′,7-hexahydroxyflavanone, philorizin, and trilobatin could play crucial roles in influencing the antioxidant potential of black rice kernels.

## Figures and Tables

**Table 1:** Differences in physiological and biochemical indices of colored rice grains.

Pathway Description	Pathway_ID	Metabolites	*p* Value
KEGG pathways between YZ6H and YJN818			
Citrate cycle (TCA cycle)	map00020	C00158; C00311; C00417	0.0016
Phenylalanine, tyrosine and tryptophan biosynthesis	map00400	C00251; C00944; C00493	0.0074
Anthocyanin biosynthesis	map00942	C05905; C05904; C05908; C08639	0.0075
Flavone and flavonol biosynthesis	map00944	C10107; C00389; C01750; C01514; C01265; C05623	0
Flavonoid biosynthesis	map00941	C10107; C09727; C06562; C00389; C05631; C01709; C01514; C05908; C05905; C05904; C00774	0
KEGG pathways between YZ6H and HN			
Anthocyanin biosynthesis	map00942	C05905; C05908; C08639	0.0597
Flavone and flavonol biosynthesis	map00944	C10107; C00389; C01750; C01514; C01265; C05623	0.0001
Flavonoid biosynthesis	map00941	C10107; C09727; C00389; C05631; C01709; C00774; C01514; C05908; C05905; C01604; C12127; C12136	0
KEGG pathways between YZ6H and YCN1H			
Phenylalanine, tyrosine and tryptophan biosynthesis	map00400	C00251; C00944; C00493	0.012
Anthocyanin biosynthesis	map00942	C05905; C12140; C05908; C08639	0.0138
Flavone and flavonol biosynthesis	map00944	C10107; C00389; C01750; C01514; C01265; C05623; C12627	0
Flavonoid biosynthesis	map00941	C10107; C09727; C06562; C00389; C05631; C01709; C00774; C01514; C05908; C05905; C01604; C12127; C12136	0
KEGG pathways between YJN818 and HN			
Anthocyanin biosynthesis	map00942	C05905	0.5967
Flavone and flavonol biosynthesis	map00944	C10107; C05623	0.1518
Phenylalanine, tyrosine and tryptophan biosynthesis	map00400	C00251; C00944; C00493	0.0106
Flavonoid biosynthesis	map00941	C10107; C05631; C05905; C12136; C12127; C00774	0.0004
Alanine, aspartate and glutamate metabolism	map00250	C00025; C00158; C00026; C00064; C00940; C00049	0
KEGG pathways between YJN818 and YCN1H			
Anthocyanin biosynthesis	map00942	C05905; C12140	0.2973
Phenylalanine, tyrosine and tryptophan biosynthesis	map00400	C00078; C00251; C00944; C00493	0.0022
Phenylpropanoid biosynthesis	map00940	C00482; C05158; C02666; C02325; C01197	0.005
Flavone and flavonol biosynthesis	map00944	C10107; C01514; C12627; C05623	0.0095
Flavonoid biosynthesis	map00941	C10107; C09727; C06562; C05631; C01709; C00774; C01514; C05905; C01604; C12127; C12136	0
KEGG pathways between HN and YCN1H			
Phenylalanine, tyrosine and tryptophan biosynthesis	map00400	C00078; C00944	0.0575
Anthocyanin biosynthesis	map00942	C12140	0.5359
Flavone and flavonol biosynthesis	map00944	C01514; C12627; C05623	0.0202
Phenylpropanoid biosynthesis	map00940	C00482; C05158; C02666; C01197; C12205	0.001
Flavonoid biosynthesis	map00941	C09727; C06562; C01514; C01709; C05631; C12136; C12127; C00774	0

Pathway Description: The name and ID of the KEGG pathway; Metabolites: Metabolites participating in the metabolic pathway. Significant enrichment terms were defined as those with a *p*-value less than 0.05.

**Table 2:** Quantitative measurement of flavonoids and phenolic compounds in grains.

Metabolites	YZ6H	YJN818	HN	YCN1H
2,4-Dihydroxybenzoic acid	153.57 ± 25.45a	128.03 ± 5.62a	138.46 ± 15.14a	152.92 ± 2.3a
2,6-Dihydroxybenzoic acid	85.49 ± 5.66c	112.67 ± 5.96ab	105.39 ± 2.42b	123.29 ± 4.7a
3,4-Dihydroxybenzaldehyde	48.12 ± 4.98c	823.57 ± 54.3a	143.67 ± 15.19b	0 ± 0c
4-Hydroxybenzoic acid	174.26 ± 14.95b	55.53 ± 8.22d	92.58 ± 6.27c	233.96 ± 1.98a
4-Hydroxycinnamic acid	1180.27 ± 16.56b	828.89 ± 36.26c	1131.57 ± 20.34b	1250.14 ± 22.18a
Apiin	5.9 ± 0.34b	11.06 ± 1.25a	11.29 ± 1.18a	11.81 ± 1.54a
Aromadendrin	16.81 ± 0.36a	13.24 ± 0.96b	12.37 ± 0.72b	1.42 ± 0.02c
Caffeic acid	181.23 ± 22.01b	253.5 ± 33.52b	345.82 ± 47.58a	79.02 ± 1.32c
Caftaric acid	8.55 ± 1.36c	11.06 ± 0.47bc	28.61 ± 3.04a	13.84 ± 0.85b
Catechin	35.51 ± 2.69c	9363.44 ± 357.32a	3681.15 ± 195.08b	0 ± 0c
Chlorogenic acid	8.94 ± 0.56a	0 ± 0b	0 ± 0b	0 ± 0b
Coniferaldehyde	0 ± 0b	0 ± 0b	0 ± 0b	2.07 ± 0.42a
Cosmosiin	12.92 ± 0.88a	13.25 ± 0.28a	4.52 ± 0.38b	3.06 ± 0.21b
Cyanidin 3-O-rutinoside chloride	17,133.01 ± 669.36a	24.97 ± 2.85b	0 ± 0b	6.16 ± 1.43b
Epicatechin	0 ± 0b	53.35 ± 5.88a	0 ± 0b	0 ± 0b
Eriodictyol	25.38 ± 1.19a	8.36 ± 0.29b	1.7 ± 0.34c	0 ± 0c
Ferulic acid	1655.96 ± 70.62d	2075.78 ± 50.11c	3901.8 ± 101.15b	4441.52 ± 127.71a
Gallic acid	74.28 ± 1.63a	4.38 ± 0.19b	3.27 ± 0.42b	3 ± 0.47b
Gentisic acid	81.9 ± 7.35a	46.04 ± 8.89b	47.6 ± 5.8b	22.64 ± 1.51c
Hesperidin	125.36 ± 12.51a	6.33 ± 1.46b	4.75 ± 0.23b	4.96 ± 1.04b
Isoorientin	860.2 ± 17.39a	184.69 ± 9.25b	121.38 ± 7.86c	19.55 ± 1.82d
Isorhamnetin	2.2 ± 0.13a	0.45 ± 0.02b	0 ± 0c	0 ± 0c
Isorhamnetin-3-O-glucoside	1008.38 ± 20.38a	69.4 ± 6.45b	29.06 ± 1.63c	3.35 ± 0.13c
Methyl gallate	136.84 ± 2.98a	2.28 ± 0.23b	0.35 ± 0.04b	0.48 ± 0.05b
Morin	11.59 ± 0.52a	0.62 ± 0.03b	0 ± 0b	0 ± 0b
Myricetin 3-galactoside	343.38 ± 16.36a	0 ± 0b	0 ± 0b	0 ± 0b
Narcissin	346.68 ± 16.05a	4.62 ± 0.56b	1.44 ± 1.05b	3.42 ± 0.78b
Naringenin	11.3 ± 0.51b	48.79 ± 2.09a	14.16 ± 0.66b	0 ± 0c
Naringin	19.64 ± 3.14a	0 ± 0b	0 ± 0b	0 ± 0b
Nicotiflorin	4.96 ± 0.4a	0 ± 0c	1.12 ± 0.16b	0 ± 0c
Orientin	39.5 ± 1.1a	2.82 ± 0.29b	1.83 ± 0.06b	0 ± 0c
Pelargonidin-3-glucoside	1878.84 ± 127.67a	0 ± 0b	0 ± 0b	0 ± 0b
Phloretin	0.38 ± 0.01a	0 ± 0b	0 ± 0b	0 ± 0b
Phlorizin	148.86 ± 8.03a	0 ± 0b	0 ± 0b	0 ± 0b
Procyanidin B3	41.87 ± 5.16c	39,397.65 ± 3851.68a	11,397.62 ± 860.57b	0 ± 0c
Protocatechuic acid	21,480.88 ± 1200.16a	1052.57 ± 8.97b	184.97 ± 14.37b	43.4 ± 1.56b
Prunin	34.77 ± 4.68a	0.82 ± 0.14b	0 ± 0b	0 ± 0b
Quercetin 3-galactoside	5969.76 ± 294.12a	39.62 ± 4.34b	12.78 ± 1.65b	0 ± 0b
Quercetin	11.66 ± 0.47a	0.52 ± 0.03b	0 ± 0b	0 ± 0b
Rutin	1388.95 ± 155a	3.96 ± 0.35b	0 ± 0b	3.05 ± 0.31b
Salicylic acid	1174.88 ± 16.27a	672.46 ± 9.05b	588.47 ± 20.49c	508.82 ± 16.92d
Sinapic acid	995.84 ± 15.07a	420.6 ± 14.87c	768.46 ± 15.07b	269.58 ± 5.62d
Syringaldehyde	39.65 ± 2.78a	29.15 ± 1.46b	37 ± 2.83a	43.56 ± 2.19a
Syringic acid	152.78 ± 27.37a	65.3 ± 8.39b	66.3 ± 5.02b	50.12 ± 4.59b
Taxifolin	3997.79 ± 120.18a	445 ± 19.93b	146.86 ± 9.25c	0 ± 0c
trans-3,3′,4′,5,5′,7-Hexahydroxyflavanone	228.27 ± 18.71a	0 ± 0b	0 ± 0b	0 ± 0b
trans-Cinnamic acid	37.56 ± 2.33c	62.54 ± 2.44a	68.33 ± 3.57a	48.7 ± 1.18b
trans-Piceid	336.9 ± 10.03a	11.67 ± 0.47b	4.03 ± 0.55b	0 ± 0b
Trilobatin	1.04 ± 0.04a	0 ± 0b	0 ± 0b	0 ± 0b
Vanillic acid	8942.13 ± 407.93a	156.65 ± 10.53b	272.31 ± 27.35b	247.69 ± 25.61b
Vitexin	150.11 ± 14.69a	67.62 ± 6.22b	39.84 ± 5.21c	18.53 ± 1.12c

The measurement unit is (ng g^−1^). Different lowercase letters on the same line represent the significance of *p* values at the 0.05 level.

**Table 3:** Correlation analysis of physiological and biochemical indices and flavonoid contents.

Index	CAT	POD	SOD	PPO	PAL	TP	Flavonoids	OPC	ABTS	DPPH	FRAP
2,4-Dihydroxybenzoic acid	0.732 **	0.51	0.678 *	0.018	0.314	0.261	0.3	0.235	0.254	0.26	0.232
2,6-Dihydroxybenzoic acid	−0.157	−0.271	−0.36	−0.352	−0.797 **	−0.717 **	−0.791 **	−0.686 *	−0.849 **	−0.835 **	−0.843 **
3,4-Dihydroxybenzaldehyde	−0.259	−0.863 **	−0.691 *	−0.586 *	−0.143	−0.09	−0.188	−0.048	−0.18	−0.172	−0.144
4-Hydroxybenzoic acid	0.428	0.393	0.471	0.099	0.149	0.226	0.237	0.213	0.161	0.178	0.15
4-Hydroxycinnamic acid	0.265	0.812 **	0.699 *	0.536	0.09	0.059	0.141	0.026	0.118	0.114	0.083
Apiin	−0.288	−0.182	−0.525	−0.161	−0.897 **	−0.872 **	−0.910 **	−0.853 **	−0.914 **	−0.919 **	−0.921 **
Aromadendrin	−0.076	−0.038	0.083	0.128	0.655 *	0.538	0.585 *	0.518	0.667 *	0.647 *	0.667 *
Caffeic acid	−0.436	0.082	−0.176	0.342	−0.18	−0.314	−0.252	−0.327	−0.142	−0.174	−0.153
Caftaric acid	−0.404	0.56	−0.037	0.618 *	−0.613 *	−0.763 **	−0.664 *	−0.793 **	−0.585 *	−0.614 *	−0.611 *
Catechin	−0.394	−0.732 **	−0.718 **	−0.434	−0.301	−0.283	−0.358	−0.248	−0.328	−0.329	−0.3
Chlorogenic acid	0.405	0.247	0.545	0.145	0.962 **	0.927 **	0.973 **	0.899 **	0.980 **	0.978 **	0.971 **
Coniferaldehyde	0.152	0.079	0.089	−0.159	−0.399	−0.278	−0.325	−0.262	−0.412	−0.396	−0.425
Cosmosiin	0.06	−0.577 *	−0.151	−0.386	0.706 *	0.735 **	0.683 *	0.751 **	0.694 *	0.699 *	0.718 **
Cyanidin 3−O−rutinoside chloride	0.37	0.245	0.583 *	0.164	0.965 **	0.929 **	0.975 **	0.901 **	0.983 **	0.981 **	0.975 **
Epicatechin	−0.233	−0.920 **	−0.674 *	−0.658 *	−0.113	−0.029	−0.144	0.019	−0.155	−0.142	−0.117
Eriodictyol	0.351	−0.011	0.374	−0.032	0.985 **	0.961 **	0.977 **	0.946 **	0.987 **	0.987 **	0.989 **
Ferulic acid	−0.133	0.431	0.003	0.287	−0.801 **	−0.803 **	−0.773 **	−0.810 **	−0.788 **	−0.0792 **	−0.810 **
Gallic acid	0.389	0.235	0.562	0.142	0.970 **	0.933 **	0.978 **	0.906 **	0.985 **	0.983 **	0.977 **
Gentisic acid	0.036	0.127	0.365	0.293	0.847 **	0.774 **	0.830 **	0.749 **	0.887 **	0.871 **	0.879 **
Hesperidin	0.425	0.266	0.606 *	0.129	0.976 **	0.926 **	0.971 **	0.899 **	0.973 **	0.972 **	0.964 **
Isoorientin	0.341	0.163	0.496	0.11	0.984 **	0.934 **	0.975 **	0.909 **	0.992 **	0.988 **	0.987 **
Isorhamnetin	0.352	0.073	0.499	0.028	0.995 **	0.968 **	0.992 **	0.949 **	0.996 **	0.997 **	0.995 **
Isorhamnetin-3-O-glucoside	0.37	0.216	0.566	0.137	0.979 **	0.938 **	0.981 **	0.912 **	0.990 **	0.988 **	0.983 **
Methyl gallate	0.402	0.247	0.580 *	0.141	0.974 **	0.933 **	0.978 **	0.905 **	0.983 **	0.981 **	0.975 **
Morin	0.381	0.214	0.582 *	0.125	0.981 **	0.941 **	0.983 **	0.916 **	0.988 **	0.987 **	0.982 **
Myricetin 3-galactoside	0.384	0.243	0.558	0.156	0.962 **	0.928 **	0.974 **	0.901 **	0.982 **	0.980 **	0.974 **
Narcissin	0.389	0.237	0.555	0.15	0.964 **	0.931 **	0.976 **	0.904 **	0.983 **	0.981 **	0.975 **
Naringenin	−0.306	−0.804 **	−0.624 *	−0.484	−0.022	0.012	−0.074	0.051	−0.052	−0.05	−0.021
Naringin	0.383	0.221	0.483	0.147	0.938 **	0.915 **	0.959 **	0.888 **	0.969 **	0.966 **	0.961 **
Nicotiflorin	0.344	0.393	0.574	0.306	0.903 **	0.828 **	0.904 **	0.790 **	0.936 **	0.925 **	0.920 **
Orientin	0.371	0.217	0.54	0.143	0.973 **	0.933 **	0.978 **	0.907 **	0.990 **	0.987 **	0.982 **
Pelargonidin-3-glucoside	0.385	0.263	0.621 *	0.16	0.971 **	0.926 **	0.973 **	0.898 **	0.978 **	0.976 **	0.970 **
Phloretin	0.367	0.248	0.593 *	0.167	0.966 **	0.928 **	0.975 **	0.900 **	0.982 **	0.980 **	0.974 **
Phlorizin	0.371	0.255	0.610 *	0.166	0.969 **	0.927 **	0.974 **	0.899 **	0.981 **	0.979 **	0.972 **
Procyanidin B3	−0.362	−0.795 **	−0.708 *	−0.478	−0.257	−0.211	−0.303	−0.166	−0.287	−0.284	−0.258
Protocatechuic acid	0.414	0.22	0.536	0.114	0.975 **	0.938 **	0.980 **	0.911 **	0.986 **	0.984 **	0.978 **
Prunin	0.441	0.23	0.497	0.107	0.959 **	0.925 **	0.967 **	0.898 **	0.973 **	0.971 **	0.965 **
Quercetin 3-galactoside	0.411	0.249	0.554	0.14	0.967 **	0.929 **	0.974 **	0.901 **	0.981 **	0.979 **	0.972 **
Quercetin	0.365	0.211	0.572	0.139	0.975 **	0.940 **	0.982 **	0.914 **	0.989 **	0.987 **	0.982 **
Rutin	0.452	0.271	0.579 *	0.122	0.971 **	0.922 **	0.967 **	0.894 **	0.970 **	0.969 **	0.961 **
Salicylic acid	0.352	0.113	0.478	0.079	0.985 **	0.940 **	0.975 **	0.918 **	0.992 **	0.989 **	0.988 **
Sinapic acid	0.093	0.519	0.505	0.516	0.707 *	0.548	0.662 *	0.499	0.743 **	0.717 **	0.720 **
Syringaldehyde	0.309	0.603 *	0.433	0.381	0.039	0.061	0.119	0.032	0.079	0.089	0.066
Syringic acid	0.168	0.202	0.631 *	0.274	0.907 **	0.859 **	0.910 **	0.837 **	0.932 **	0.927 **	0.927 **
Taxifolin	0.384	0.179	0.514	0.103	0.982 **	0.945 **	0.984 **	0.920 **	0.994 **	0.992 **	0.988 **
trans-3,3′,4′,5,5′,7-Hexahydroxyflavanone	0.345	0.236	0.579 *	0.176	0.958 **	0.925 **	0.972 **	0.898 **	0.981 **	0.978 **	0.973 **
trans-Cinnamic acid	−0.511	−0.156	−0.576	0.034	−0.757 **	−0.812 **	−0.807 **	−0.803 **	−0.742 **	−0.761 **	−0.742 **
trans-Piceid	0.394	0.229	0.55	0.136	0.972 **	0.935 **	0.979 **	0.907 **	0.986 **	0.984 **	0.979 **
Trilobatin	0.399	0.262	0.602 *	0.153	0.972 **	0.928 **	0.975 **	0.900 **	0.980 **	0.978 **	0.971 **
Vanillic acid	0.411	0.275	0.604 *	0.154	0.970 **	0.923 **	0.972 **	0.895 **	0.977 **	0.975 **	0.968 **
Vitexin	0.261	−0.025	0.349	0.019	0.950 **	0.921 **	0.947 **	0.902 **	0.972 **	0.969 **	0.975 **

* *p* < 0.05; ** *p* < 0.01.

## Data Availability

Data are contained within the article and Supplementary Materials.

## Supplementary Materials

The following supporting information can be downloaded from https://www.mdpi.com/article/10.3390/metabo14020120/s1. Figure S1: Ion current diagram. (A) Total ion current diagram (standard), (B) negative ion current diagram, (C) positive ion current diagram, (D) total ion current diagram (QC sample superposition), (E) total ion current diagram (standard superposition). Figure S2: Statistical map of the compounds. The name and percentage of the metabolites in the selected HMDB hierarchy (Class) are displayed in order from highest to lowest, depending on the number of metabolites. The different colors in each pie plot in the figure represent different HMDB classifications, and the area represents the relative proportion of the metabolites in that classification. (A) YZ6H and YJN818, (B) YZ6H and HN, (C) YZ6H and YCN1H, (D) YJN818 and HN, (E) YJN818 and YCN1H, (F) HN and YCN1H. Figure S3: DM volcano map. Each dot represents a specific metabolite, and the size of the dot represents the VIP value. On the left are differentially abundant metabolites with lower abundance, and on the right are differentially abundant metabolites with higher abundance; the more left or right they are, the greater the difference. (A) YZ6H and YJN818, (B) YZ6H and HN, (C) YZ6H and YCN1H, (D) YJN818 and HN, (E) YJN818 and YCN1H, (F) HN and YCN1H. Table S1: Standard information on flavonoids and phenolic compounds. Table S2: Linear equations for the flavonoids and phenolic compounds. Table S3: DM information for the YZ6H and YJN818 comparison groups. Table S4: DM information for the YZ6H and HN comparison groups. Table S5: DM information for the YZ6H and YCN1H comparison groups. Table S6: DM information for the YJN818 and HN comparison groups. Table S7: DM information for the YJN818 and YCN1H comparison groups. Table S8: DM information for the HN and YCN1H comparison groups.
