# Supplementary material for: Metabolomics Reveals Antioxidant Metabolites in Colored Rice Grains
Source: Metabolites. 2024 Feb 11;14(2):120. doi: 10.3390/metabo14020120 (PMC10891847; doi:10.3390/metabo14020120)
Supplement: Supplementary file 1 [file metabolites-14-00120-s001.zip › Figure S1.pdf]

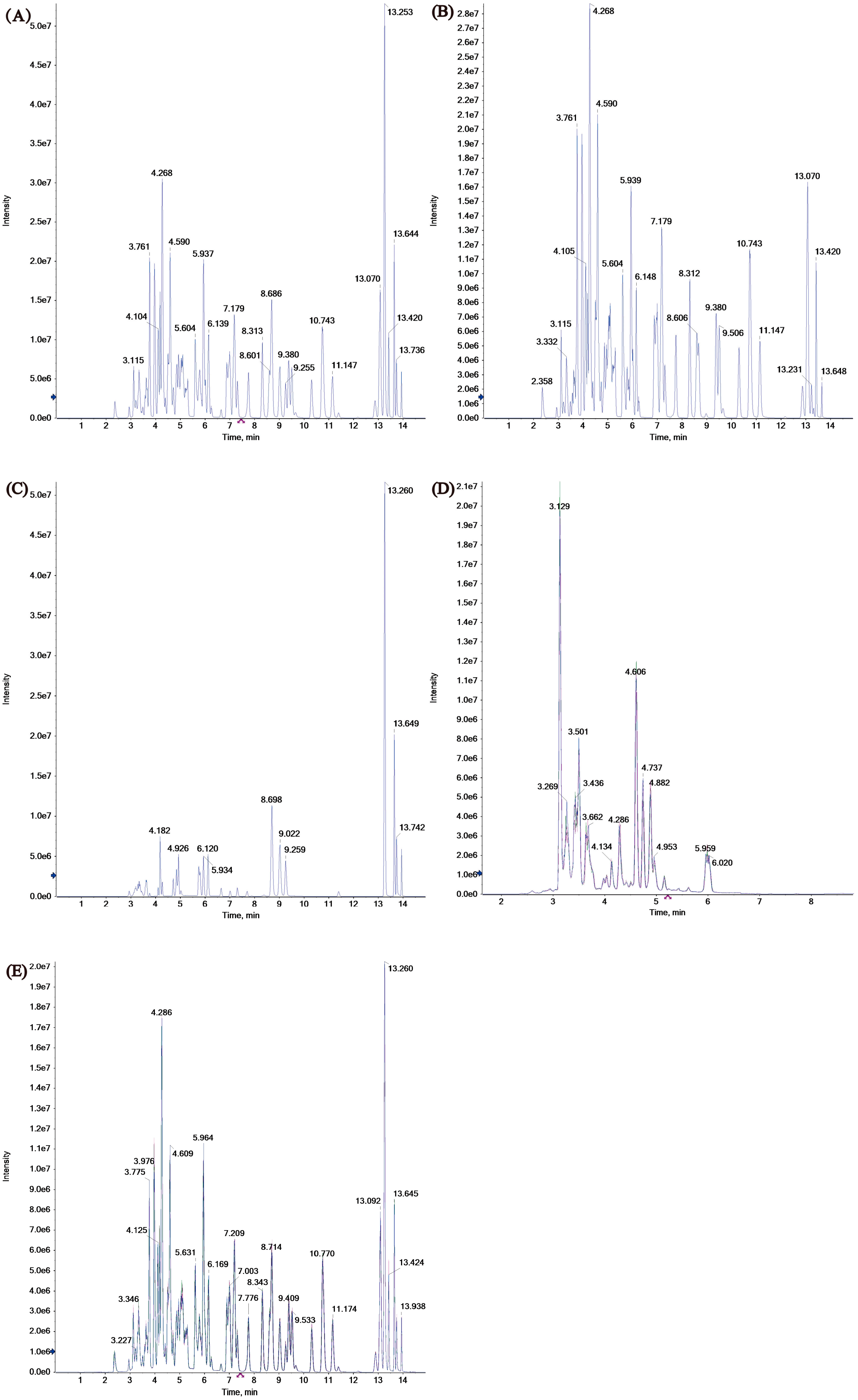
(A)
(B)
(C)
(D)
(E)
Intensity
Time, min
13.253
4.268
3.761
4.590
5.937
4.104
5.604
6.139
7.179
8.686
8.313
8.601
9.380
9.255
10.743
11.147
13.070
13.644
13.420
13.736
3.115
3.332
2.358
4.105
5.939
6.148
8.312
8.606
9.506
13.231
13.648
13.260
13.649
13.742
8.698
9.022
9.259
4.182
4.926
6.120
5.934
3.129
4.606
3.501
3.269
3.436
3.662
4.286
4.134
4.737
4.882
4.953
5.959
6.020
3.976
3.775
4.609
5.964
4.125
5.631
6.169
7.209
7.003
8.343
7.776
8.714
9.409
9.533
10.770
11.174
13.092
13.645
13.424
13.938
3.346
3.227

Figure S1. Ion current diagram. **(A)** Total ion current diagram (standard), **(B)** Extraction of negative ion current diagram, **(C)** Extraction of positive ion current diagram, **(D)** Total ion current diagram (QC sample superposition), **(E)** Total ion current diagram (standard superposition).
