# Supplementary material for: Metabolomics Reveals Antioxidant Metabolites in Colored Rice Grains
Source: Metabolites. 2024 Feb 11;14(2):120. doi: 10.3390/metabo14020120 (PMC10891847; doi:10.3390/metabo14020120)
Supplement: Supplementary file 1 [file metabolites-14-00120-s001.zip › Figure S2.pdf]

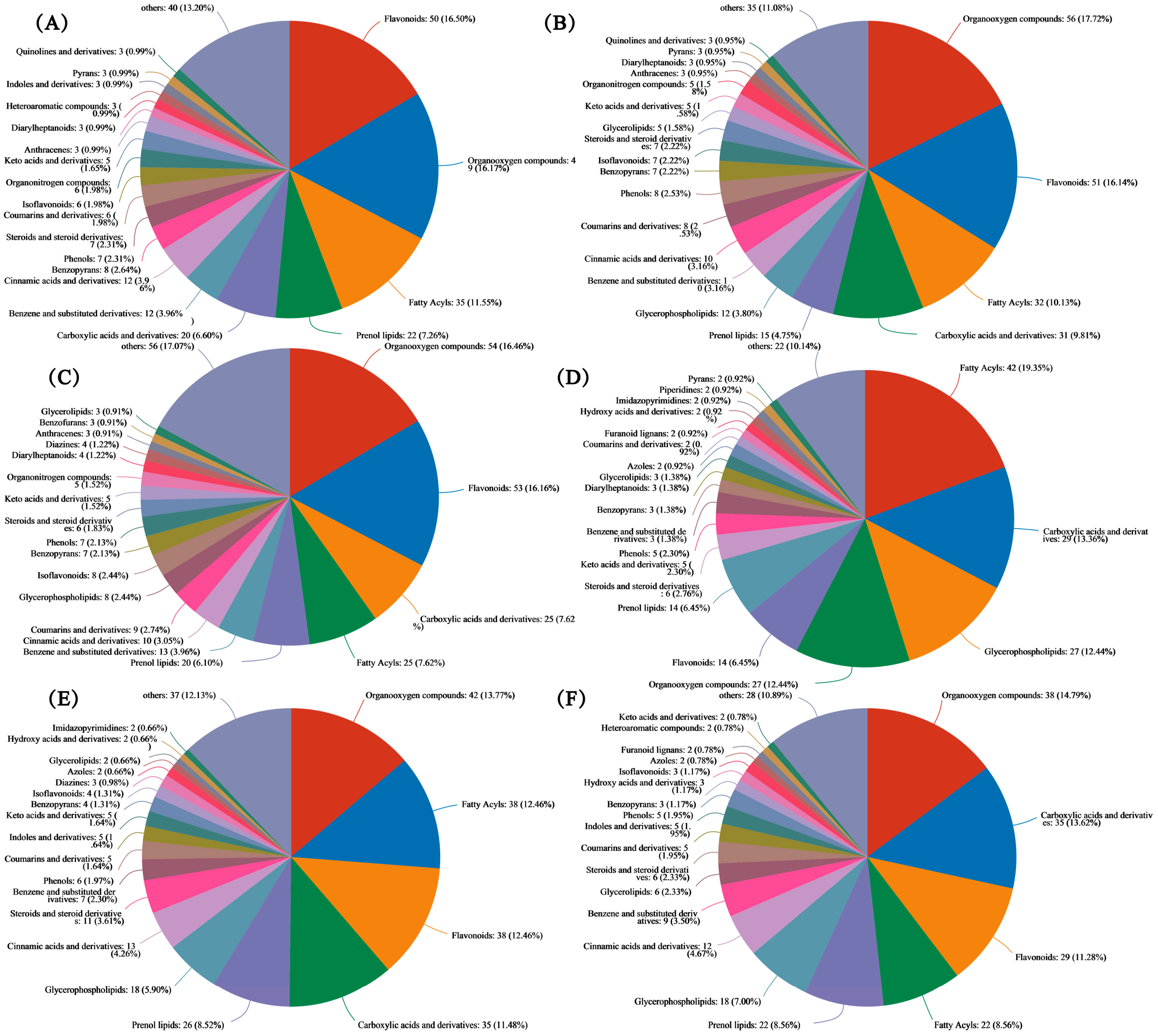

Figure S2. Statistical map of compounds. The name and percentage of the metabolites in the selected HMDB hierarchy (Class) are displayed in a high to low order, depending on the number of metabolites. The different colours in each pie plot in the figure represent a different HMDB classification, the area of which represents the relative proportion of the metabolites in that classification. **(A)** YZ6H and YJN818, **(B)** YZ6H and HN, **(C)** YZ6H and YCN1H, **(D)** YJN818 and HN,**(E)** YJN818 and YCN1H,**(F)** HN and YCN1H
