# Supplementary material for: Metabolomics Reveals Antioxidant Metabolites in Colored Rice Grains
Source: Metabolites. 2024 Feb 11;14(2):120. doi: 10.3390/metabo14020120 (PMC10891847; doi:10.3390/metabo14020120)
Supplement: Supplementary file 1 [file metabolites-14-00120-s001.zip › Figure S3.pdf]

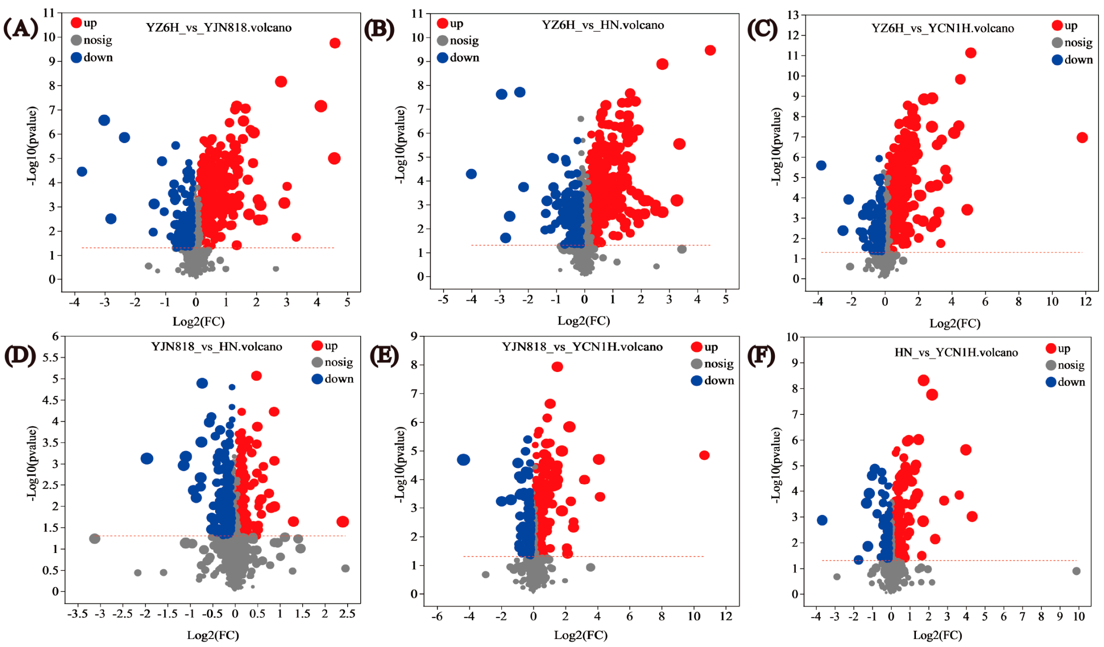

Figure S3. DM volcano map. Each dot represents a specific metabolite, and the size of the dot represents the VIP value. On the left are differentially downregulated metabolites, and on the right are differentially upregulated metabolites; the more left and right they are, the more significant the point above. (A) YZ6H and YJN818, (B) YZ6H and HN, (C) YZ6H and YCN1H, (D) YJN818 and HN, (E) YJN818 and YCN1H, (F) HN and YCN1H.
