# Supplementary material for: Metabolomics Reveals Antioxidant Metabolites in Colored Rice Grains
Source: Metabolites. 2024 Feb 11;14(2):120. doi: 10.3390/metabo14020120 (PMC10891847; doi:10.3390/metabo14020120)
Supplement: Supplementary file 1 [file metabolites-14-00120-s001.zip › Table S1.pdf]

Table S1. Standard information of flavonoids and phenolic compounds.

Name	Chemical formula	Molecular weight	Classification	Purity
(-)-Catechin 3-O-gallate	C22H18O10	442.09	Flavanols	HPLC,≥98%
(-)-Epicatechin 3-O-gallate	C22H18O10	442.09	Flavanols	HPLC,≥98%
(-)-Epigallocatechin	C15H14O7	306.07	Flavanols	HPLC,≥98%
(-)-Gallocatechin gallate	C22H18O11	458.08	Flavanols	HPLC,≥98%
(+/-)Catechin	C15H14O6	290.08	Flavanols	HPLC,≥98%
(±)-Gallocatechin	C15H14O7	306.07	Flavanols	HPLC,≥98%
(2S)-Liquiritigenin	C15H12O4	256.07	Flavanones	HPLC,≥98%
(S)-Pinocembrin	C15H12O4	256.07	Flavanones	HPLC,≥98%
1,5-Dicaffeoylquinic acid	C25H24O12	516.13	Alcohols and polyols	HPLC,≥99%
2,4-Dihydroxybenzoic acid	C7H6O4	154.03	Benzoic acid derivatives	HPLC,≥98%
2,6-Dihydroxybenzoic acid	C7H6O4	154.03	Benzoic acid derivatives	HPLC,≥98%
3,3',4'5-Tetrahydroxystilbene	C14H12O4	244.07	Stilbenes	HPLC,≥98%
3,4-Dihydro-2H-1-benzopyran-2-one	C9H8O2	148.05	Coumarins	HPLC,≥98%
3,4-Dihydroxybenzaldehyde	C7H6O3	138.03	Catechols	HPLC,≥98%
3-Hydroxybenzaldehyde	C7H6O2	122.04	Benzaldehydes	HPLC,≥95%
3-Hydroxyflavone	C15H10O3	238.06	Flavonols	HPLC,≥98%
4',7-Di-O-methylnaringenin	C17H16O5	300.10	Flavanones	HPLC,≥98%
4-Coumaryl alcohol	C9H10O2	150.07	Coumarins	HPLC,≥99%
4-Hydroxybenzoic acid	C7H6O3	138.03	Benzoic acid derivatives	HPLC,≥98%
4-Hydroxycinnamic acid	C9H8O3	164.05	Phenylpropanoids	HPLC,≥98%
4-Methylumbelliferone	C10H8O3	176.05	Coumarins	HPLC,≥98%
6-Hydroxydaidzein	C15H10O5	270.05	Isoflavones	HPLC,≥98%
6-Methoxyflavone	C16H12O3	252.08	Flavanones	HPLC,≥99.5%
6-Methylcoumarin	C10H8O2	160.05	Coumarins	HPLC,≥98%
Acetovanillone	C9H10O3	166.06	Acetophenones	HPLC,≥98%
Aesculetin	C9H6O4	178.03	Coumarins	HPLC,≥98%
Aesculin	C15H16O9	340.08	Coumarins	HPLC,≥98%
Afzelin	C21H20O10	432.11	Flavonols	HPLC,≥98%
Amentoflavone	C30H18O10	538.09	Flavones	HPLC,≥98%
Apigenin	C15H10O5	270.05	Flavones	HPLC,≥98%
Apigenin 4'-O-rhamnoside	C21H20O9	416.11	Flavonols	HPLC,≥98%
Apiin	C26H28O14	564.15	Flavones	HPLC,≥98%
Aromadendrin	C15H12O6	288.06	Flavanonols	HPLC,≥98%

Astragalin	C21H20O11	448.10	Flavonols	HPLC,≥98%
Avicularin	C20H18O11	434.08	Flavonols	HPLC,≥98%
Baicalein	C15H10O5	270.05	Flavones	HPLC,≥98%
Biochanin A	C16H12O5	284.07	Isoflavones	HPLC,≥98%
Butein	C15H12O5	272.07	Chalcones	HPLC,≥98%
Caffeic acid	C9H8O4	180.04	Phenylpropanoids	HPLC,≥98%
Caftaric acid	C13H12O9	312.05	Phenylpropanoids	HPLC,≥98%
Calycosin	C16H12O5	284.07	Isoflavones	HPLC,≥98%
Chlorogenic acid	C16H18O9	354.10	Alcohols and polyols	HPLC,≥98%
Chrysin	C15H10O4	254.06	Flavones	HPLC,≥98%
Coniferaldehyde	C10H10O3	178.06	Aldehydes	HPLC,≥98%
Cosmosiin	C21H20O10	432.11	Flavones	HPLC,≥98%
Cryptochlorogenic acid	C16H18O9	354.10	Alcohols and polyols	HPLC,≥98%
Cucurbitacin I	C30H42O7	514.29	Terpenoids	HPLC,≥98%
Cyanidin 3-O-rutinoside chloride	C27H31ClO15	630.14	Anthocyanins	HPLC,≥95%
Cyanin chloride	C27H31ClO16	646.13	Anthocyanins	HPLC,≥95%
Daidzein	C15H10O4	254.06	Isoflavones	HPLC,≥98%
Daidzin	C21H20O9	416.11	Isoflavones	HPLC,≥98%
Daphnetin	C9H6O4	178.03	Coumarins	HPLC,≥98%
Delphinidin 3-glucoside	C21H21O12	465.10	Anthocyanins	HPLC,≥95%
Diosmin	C28H32O15	608.17	Flavones	HPLC,≥95%
Ellagic acid	C14H6O8	302.01	Benzoic acid derivatives	HPLC,≥98%
Epicatechin	C15H14O6	290.08	Flavanols	HPLC,≥98%
Epigallocatechin gallate	C22H18O11	458.08	Flavanols	HPLC,≥98%
Eriodictyol	C15H12O6	288.06	Flavanones	HPLC,≥98%
Ferulic acid	C10H10O4	194.06	Phenylpropanoids	HPLC,≥98%
Formononetin	C16H12O4	268.07	Isoflavones	HPLC,≥98%
Fraxetin	C10H8O5	208.04	Coumarins	HPLC,≥98%
Fraxin	C16H18O10	370.09	Coumarins	HPLC,≥98%
Galangin	C15H10O5	270.05	Flavones	HPLC,≥98%
Gallic acid	C7H6O5	170.02	Benzoic acid derivatives	HPLC,≥98%
Genistein	C15H10O5	270.05	Isoflavones	HPLC,≥98%
Gentisic acid	C7H6O4	154.03	Benzoic acid derivatives	HPLC,≥98%
Glycitein	C16H12O5	284.07	Isoflavones	HPLC,≥98%
Hesperetin	C16H14O6	302.08	Flavanones	HPLC,≥98%
Hesperidin	C28H34O15	610.19	Flavanones	HPLC,≥98%
Isoliquiritigenin	C15H12O4	256.07	Chalcones	HPLC,≥98%
Isoorientin	C21H20O11	448.10	Flavones	HPLC,≥98%
Isorhamnetin	C16H12O7	316.06	Flavonols	HPLC,≥98%
Isorhamnetin-3-O-glucoside	C22H22O12	478.11	Flavonols	HPLC,≥98%
Isosakuranetin	C16H14O5	286.08	Flavanones	HPLC,≥98%

Jaceosidin	C17H14O7	330.07	Isoflavones	HPLC,≥98%
Kaempferol	C15H10O6	286.05	Flavonols	HPLC,≥98%
Lcariin	C33H40O15	676.24	Flavonols	HPLC,≥98%
Limonin	C26H30O8	470.19	Terpenoids	HPLC,≥98%
Luteolin	C15H10O6	286.05	Flavones	HPLC,≥98%
Malvin	C29H35O17	655.19	Anthocyanins	HPLC,≥95%
m-Coumaric acid	C9H8O3	164.05	Phenylpropanoids	HPLC,≥98%
Methyl gallate	C8H8O5	184.04	Benzoic acid derivatives	HPLC,≥98%
Morin	C15H10O7	302.04	Flavonols	HPLC,≥98%
Myricetin	C15H10O8	318.04	Flavonols	HPLC,≥98%
Myricetin 3-galactoside	C21H20O13	480.09	Flavonols	HPLC,≥98%
Myricitrin	C21H20O12	464.10	Flavonols	HPLC,≥98%
Narcissin	C28H32O16	624.17	Flavonols	HPLC,≥98%
Naringenin	C15H12O5	272.07	Flavanones	HPLC,≥98%
Naringin	C27H32O14	580.18	Flavanones	HPLC,≥98%
Nicotiflorin	C27H30O15	594.16	Flavonols	HPLC,≥98%
Nobiletin	C21H22O8	402.13	Flavones	HPLC,≥98%
Ononin	C22H22O9	430.13	Isoflavones	HPLC,≥98%
Orientin	C21H20O11	448.10	Flavones	HPLC,≥98%
Pelargonidin-3-glucoside	C21H21ClO1 0	468.08	Anthocyanins	HPLC,≥95%
Perillyl Alcohol	C10H16O	152.12	Terpenoids	HPLC,≥98%
Phloretin	C15H14O5	274.08	Dihydrochalcones	HPLC,≥98%
Phlorizin	C21H24O10	436.14	Dihydrochalcones	HPLC,≥98%
Proanthocyanidin A2	C30H24O12	576.13	Proanthocyanidins	HPLC,≥98%
Procyanidin B1	C30H26O12	578.14	Proanthocyanidins	HPLC,≥95%
Procyanidin B2	C30H26O12	578.14	Proanthocyanidins	HPLC,≥98%
Procyanidin B3	C30H26O12	578.14	Proanthocyanidins	HPLC,≥98%
Protocatechuic acid	C7H6O4	154.03	Benzoic acid derivatives	HPLC,≥98%
Prunin	C21H22O10	434.12	Flavonols	HPLC,≥98%
Psoralen	C11H6O3	186.03	Coumarins	HPLC,≥98%
Pterostilbene	C16H16O3	256.11	Stilbenes	HPLC,≥98%
Quercetin	C15H10O7	302.04	Flavonols	HPLC≥98%
Quercetin 3-galactoside	C21H20O12	464.10	Flavonols	HPLC,≥98%
Quercetin 3-O-glucuronide	C21H18O13	478.07	Flavonols	HPLC,≥95%
Quercitrin	C21H20O11	448.10	Flavonols	HPLC,≥98%
Resveratrol	C14H12O3	228.08	Stilbenes	HPLC,≥98%
Rhamnetin	C16H12O7	316.06	Flavonols	HPLC,≥98%
Rutin	C27H30O16	610.15	Flavonols	HPLC,≥98%
Sakuranetin	C16H14O5	286.08	Flavanones	HPLC,≥98%
Salicin	C13H18O7	286.11	Benzyl alcohols	HPLC,≥98%
Salicylic acid	C7H6O3	138.03	Benzoic acid derivatives	HPLC,≥98%
Scopoletin	C10H8O4	192.04	Coumarins	HPLC,≥99%

Sinapaldehyde	C11H12O4	208.07	Aldehydes	HPLC,≥95%
Sinapic acid	C11H12O	224.07	Phenylpropanoids	HPLC,≥98%
Syringaldehyde	C9H10O4	182.06	Benzaldehydes	HPLC,≥98%
Syringic acid	C9H10O5	198.05	Benzoic acid derivatives	HPLC,≥99%
Taxifolin	C15H12O7	304.06	Flavanonols	HPLC,≥98%
trans-3,3',4',5,5',7-Hexahydroxyflavanone	C15H12O8	320.05	Flavanonols	HPLC,≥98%
trans-Cinnamic acid	C9H8O2	148.05	Phenylpropanoids	HPLC,≥98%
trans-Piceid	C20H22O8	390.13	Stilbenes	HPLC,≥98%
Trilobatin	C21H24O10	436.14	Dihydrochalcones	HPLC,≥98%
Umbelliferone	C9H6O3	162.03	Coumarins	HPLC,≥98%
Vanillic acid	C8H8O4	168.04	Benzoic acid derivatives	HPLC,≥97%
Vanillin	C8H8O3	152.05	Benzaldehydes	HPLC,≥98%
Vitexin	C21H20O10	432.11	Flavones	HPLC,≥98%
