# Supplementary material for: Metabolomics Reveals Antioxidant Metabolites in Colored Rice Grains
Source: Metabolites. 2024 Feb 11;14(2):120. doi: 10.3390/metabo14020120 (PMC10891847; doi:10.3390/metabo14020120)
Supplement: Supplementary file 1 [file metabolites-14-00120-s001.zip › Table S2.pdf]

Table S2. The linear equations for the flavonoids and phenolic compounds.

Metabolites	Retention time	limit of detection	limit of quantitation	Standard curves	RSD (%)
2,4-Dihydroxybenzoic acid	4.28	4.27	4.27	y = 1.66737e5 x + 5.30965e5 (r = 0.99751, r^2 = 0.99503)	1.76
2,6-Dihydroxybenzoic acid	4.28	0.14	0.14	y = 8.86564e4 x + 6476.54125 (r = 0.99969, r^2 = 0.99939)	3.32
3,4-Dihydroxybenzaldehyde	3.68	0.69	0.69	y = 22116.62642 x + 1050.00105 (r = 0.99923, r^2 = 0.99846)	7.62
4-Hydroxybenzoic acid	3.75	1.56	1.90	y = 3.88878e4 x + -11616.14538 (r = 0.99960, r^2 = 0.99920)	7.81
4-Hydroxycinnamic acid	4.59	0.12	0.77	y = 9.73960e4 x + 3.26284e4 (r = 0.99940, r^2 = 0.99879)	5.35
Apiin	4.93	0.04	0.04	y = 27062.60184 x + 1005.19708 (r = 0.99730, r^2 = 0.99461)	3.91
Aromadendrin	5.85	0.26	0.26	y = 4.73204e4 x + -214.31868 (r = 0.99952, r^2 = 0.99903)	4.59
Caffeic acid	3.96	1.30	2.10	y = 26134.20850 x + -3.03375e4 (r = 0.99590, r^2 = 0.99181)	15.71
Caftaric acid	3.24	0.38	0.97	y = 7984.42499 x + -1080.86629 (r = 0.99809, r^2 = 0.99618)	3.55
Catechin	3.63	1.21	0.32	y = 4196.27171 x + -2962.01711 (r = 0.99873, r^2 = 0.99745)	5.85
Chlorogenic acid	3.58	0.28	0.28	y = 24371.86981 x + -3680.26502 (r = 0.99975, r^2 = 0.99950)	3.09
Coniferaldehyde	5.75	0.20	0.71	y = 3.57501e4 x + 6488.86865 (r = 0.99881, r^2 = 0.99761)	2.85
Cosmosiin	5.20	0.12	0.27	y = 22223.67911 x + 315.82508 (r = 0.99901, r^2 = 0.99803)	6.24
Cyanidin 3-O-rutinoside chloride	3.44	0.23	0.90	y = 14926.94965 x + 3174.56338 (r = 0.99990, r^2 = 0.99981)	9.44
Epicatechin	3.97	4.41	4.41	y = 1959.21571 x + 6949.77109 (r = 0.99695, r^2 = 0.99390)	6.71
Eriodictyol	6.95	0.29	0.12	y = 5.40788e4 x + 2823.21028 (r = 0.99952, r^2 = 0.99903)	2.06
Ferulic acid	4.88	0.37	0.37	y = 25482.04665 x + 2749.52295 (r = 0.99945, r^2 = 0.99891)	2.44
Gallic acid	2.39	0.36	0.68	y = 3.79671e4 x + 2229.43613 (r = 0.99911, r^2 = 0.99822)	0.72
Gentisic acid	3.77	2.29	2.29	y = 17580.23138 x + 25022.93985 (r = 0.99953, r^2 = 0.99906)	2.17
Hesperidin	5.21	0.05	0.05	y = 22005.34237 x + 1131.80631 (r = 0.99969, r^2 = 0.99939)	4.29

Isoorientin	4.13	0.17	0.31	y = 18849.93508 x + 162.14385 (r = 0.99947, r^2 = 0.99895)	13.67
Isorhamnetin	9.39	0.04	0.06	y = 1.51689e5 x + -855.62356 (r = 0.99987, r^2 = 0.99975)	2.66
Isorhamnetin-3-O-glucoside	5.14	0.02	0.06	y = 4.16905e4 x + 633.48862 (r = 0.99963, r^2 = 0.99925)	2.55
Methyl gallate	3.77	0.01	0.05	y = 9.68578e4 x + 797.63243 (r = 0.99903, r^2 = 0.99807)	9.56
Morin	7.20	0.05	0.05	y = 9.51372e4 x + -225.47030 (r = 0.99994, r^2 = 0.99987)	3.72
Myricetin 3-galactoside	4.19	0.09	0.30	y = 4.30538e4 x + -2095.68184 (r = 0.99857, r^2 = 0.99714)	12.18
Narcissin	4.83	0.06	0.81	y = 23437.80544 x + 6991.94109 (r = 0.99607, r^2 = 0.99215)	2.11
Naringenin	8.57	0.04	0.13	y = 14751.12788 x + 156.86294 (r = 0.99939, r^2 = 0.99878)	2.91
Naringin	5.02	0.77	0.77	y = 10536.68448 x + 3429.94710 (r = 0.99924, r^2 = 0.99847)	7.99
Nicotiflorin	4.74	0.06	0.13	y = 26923.85137 x + -297.35940 (r = 0.99926, r^2 = 0.99852)	6.39
Orientin	4.13	0.17	0.12	y = 17897.20754 x + -198.05497 (r = 0.99836, r^2 = 0.99673)	8.87
Pelargonidin-3-glucoside	3.63	0.34	1.95	y = 5.01874e4 x + 29657.21309 (r = 0.99962, r^2 = 0.99923)	7.46
Phloretin	8.61	0.11	0.11	y = 8.49143e4 x + 1171.43150 (r = 0.99972, r^2 = 0.99944)	2.42
Phlorizin	5.61	0.03	0.04	y = 4.76082e4 x + 48.26600 (r = 0.99993, r^2 = 0.99985)	4.92
Procyanidin B3	3.50	0.48	1.99	y = 2672.61615 x + -882.49941 (r = 0.99814, r^2 = 0.99627)	6.76
Protocatechuic acid	3.13	0.34	0.66	y = 3.91198e4 x + 8202.36981 (r = 0.99962, r^2 = 0.99923)	1.60
Prunin	5.25	0.02	0.05	y = 3.93228e4 x + 785.47821 (r = 0.99969, r^2 = 0.99939)	1.53
Quercetin 3-galactoside	4.55	0.19	0.34	y = 19740.06507 x + -2053.94721 (r = 0.99842, r^2 = 0.99684)	4.50
Quercetin	4.55	0.19	0.04	y = 8.82925e4 x + 1234.95714 (r = 0.99969, r^2 = 0.99938)	2.17
Rutin	4.41	0.20	0.90	y = 16280.02981 x + 403.51215 (r = 0.99540, r^2 = 0.99083)	9.51
Salicylic acid	6.01	0.01	0.74	y = 6.48909e4 x + 21122.80594 (r = 0.99885, r^2 = 0.99769)	2.71
Sinapic acid	4.86	0.04	0.04	y = 17731.96607 x + 180.36652 (r = 0.99911, r^2 = 0.99823)	5.32

Syringaldehyde	4.86	0.79	0.79	y = 19987.05676 x + 5280.46714 (r = 0.99845, r² = 0.99691)	5.83
Syringic acid	4.11	1.57	1.86	y = 7278.24918 x + -1701.88282 (r = 0.99568, r² = 0.99137)	9.31
Taxifolin	4.94	0.18	0.28	y = 20378.10557 x + 1358.56780 (r = 0.99952, r² = 0.99904)	0.76
trans-3,3',4',5,5',7-Hexahydroxyflavanone	4.18	0.12	0.12	y = 25468.90594 x + 2161.50341 (r = 0.99914, r² = 0.99828)	3.02
trans-Cinnamic acid	7.70	0.91	0.91	y = 12408.26702 x + 6154.59698 (r = 0.99995, r² = 0.99990)	2.89
trans-Piceid	4.55	0.28	0.28	y = 22325.86508 x + 2290.94663 (r = 0.99607, r² = 0.99216)	4.68
Trilobatin	6.16	0.02	0.02	y = 4.10956e4 x + 398.33363 (r = 0.99980, r² = 0.99961)	5.11
Vanillic acid	4.03	0.81	4.84	y = 1714.08337 x + 3980.22011 (r = 0.99783, r² = 0.99566)	4.86
Vitexin	4.50	0.02	0.05	y = 6.32078e4 x + -136.09591 (r = 0.99910, r² = 0.99820)	6.80
